# Around the Hospitals

**Published:** 1892-08-06

**Authors:** 


### Around the Hospitals.

The **Belfast Hospital for Diseases of the Skin** is about to make fresh efforts to increase the funds of the institution, by forming a Ladies' Committee and appointing local secretaries throughout Ulster. The reason for creating the last-named offices is the entire absence of contributions from districts sending patients to the hospital. As one-third of the patients benefited have been sent from the country, it is but just that some recognition should be received by the institution from this source. The number of patients received as in and out patients during the past year was 1,166. These figures are about the same as those of the previous year. The hospital is economically and carefully managed, but owing to the falling-off of contributions, the income for the year is insufficient.

The **London Skin Hospital** held its fifth annual meeting at the hospital, 40, Fitzroy Square, on the 30th ult. The chief features of interest in the report read on the occasion was the increase in the number of out-patients, and the fact that the Committee had been able to open in March last their much-needed in-patients' department for paying patients. There is, however, a debt of £500 incurred chiefly by reason of the heavy expenses in connection with the alterations and improvements in the new premises in Fitzroy Square, and the furnishing and fitting up of the out and in patients' departments, which the Committee would be glad to be in a position to defray, and for which they ask for funds. The re-election of the President (the Marquis of Carmarthen) and other officers took place at the meeting. The Committee wish it to be generally known that they are prepared to receive in-patients into the hospital upon terms which can be ascertained upon application to the Secretary, 40, Fitzroy Square, W.

The **Hospital for Epilepsy and Paralysis, Regent's Park,** is entirely without endowment, and annual subscriptions do not amount to £500. The average annual expenditure is about £2,000, whilst the receipts amount to little over £1,000. So large a yearly deficit is a serious responsibility to the management, and can only be obtained by strenuous efforts on their part. Epileptic and paralytic cases are those above all others suitable for admission into a hospital, where alone, owing to their frequently entire helplessness, they can receive the attention they require. For this reason a special appeal for assistance for such an institution should not be passed over. Some of the patients have to be retained within the hospital for long periods. During 1891 the number of in-patients amounted to 122. There was a slight increase in annual subscriptions during the year; funds from other sources were slightly less. The hospital, in conjunction with most other metropolitan medical charities, has adopted the uniform statement of accounts now used by the Hospital Sunday Fund, and which was prepared and approved of by a special committee of hospital secretaries during the present year.

The **Bradford Infirmary** is the centre of a scheme of sick relief of great completeness and efficiency. During the past year the important addition of the Woodlands Convalescent Home has been made, in conformity with a trust deed made by the late Sir H. Ripley, the founder. The whole neighbourhood will benefit through this munificent legacy. Since entering into possession the Infirmary Board have caused the whole system of drainage and of sanitary arrangements to be overhauled, and have also purchased additional furniture—undertakings which have involved a large expenditure—but this, having regard to the increased advantages afforded to the public by the acquisition of the Home, will doubtless be defrayed by larger donations and subscriptions. The falling-off in legacies, donations, and subscriptions during the past year, which caused a diminution of £2,659, as compared with the previous year's contributions, is a very serious loss, and one we trust that will not occur again. The hospital accounts show a balance to the good, but this, with an increased sphere of action cannot continue without increased liberality on the part of the public. During the year the heating apparatus of the large medical wards, and the ground floor corridor, has been renewed, and a second operation room has been arranged.

